# Resection of the urinary bladder for locally advanced colorectal cancer: a retrospective comparison of partial versus total cystectomy

**DOI:** 10.1186/s12893-019-0522-8

**Published:** 2019-06-17

**Authors:** Akihiro Kondo, Takeshi Sasaki, Daichi Kitaguchi, Yuichiro Tsukada, Yuji Nishizawa, Masaaki Ito

**Affiliations:** Department of Colorectal Surgery, National Cancer Center Hospital East, 6-5-1 Kashiwanoha, Kashiwa, Chiba, 277-8577 Japan

**Keywords:** Colorectal cancer, Cystectomy, Urinary bladder, Multivisceral resection

## Abstract

**Background:**

The postoperative and survival outcomes of patients with primary advanced colorectal cancer who undergo partial versus total cystectomy have not been adequately compared, as studies of this topic are rare and comprise relatively small cohorts. This study aimed to investigate the short- and long-term outcomes of patients who underwent en bloc partial or total cystectomy for primary advanced colorectal cancer that was suspected of adhering to or invading the urinary bladder.

**Methods:**

The study included 90 patients who underwent various degrees of cystectomy between 1993 and 2013 to treat locally advanced primary colorectal cancer that was suspected of involving the urinary bladder. Patients in whom total cystectomy was performed solely because of prostate-invading lower rectal cancer were excluded. Data on patient characteristics and their short- and long-term outcomes were collected retrospectively to evaluate differences between partial cystectomy (the P group; *n* = 72) and total cystectomy (the T group; *n* = 18). Postoperative and oncologic outcomes were also analyzed.

**Results:**

The T group had significantly greater operating times than the P group (median, 572 vs. 346 min); blood loss volume was also greater in the T group (median, 3092 vs. 1112 mL). The postoperative overall complication rate was significantly greater in the T group than in the P group (94.4% vs. 51.4%). With a median follow-up duration of 62 months, local recurrences were observed in 22.2 and 6.9% of patients in the T and P groups, respectively. On multivariate Cox regression analyses using partial cystectomy as the reference, total cystectomy was independently associated with poorer local recurrence-free survival (hazard ratio [HR], 4.0 95% confidence interval [CI], 1.1–15.0), relapse-free survival (HR, 2.9; 95% CI, 1.2–6.9), and overall survival (HR, 2.1; 95% CI, 1.0–4.3).

**Conclusions:**

Patients who undergo en bloc total cystectomy for locally advanced colorectal cancers have worse postoperative and oncologic outcomes than those who undergo partial cystectomy.

## Background

Locally advanced colorectal tumors sometimes adhere to or invade adjacent organs, which renders multivisceral resection technically difficult to accomplish. Nevertheless, it has been reported that T4 stage per se is not a risk factor for worse long-term outcomes after curative multivisceral resection [1, 2].

Among the adjacent organs, tumors of the sigmoid colon or upper rectum most commonly adhere to or invade the urinary bladder [3]. Because it is usually difficult to determine whether the tumor is merely adhering to the urinary bladder or invading it, patients with bladder involvement undergo en bloc resection to ensure a clear surgical margin [4, 5]. Previous investigations of the outcomes of en bloc urinary bladder resection in patients with advanced colorectal cancer found that the rates of postoperative complications, local recurrence, and 5-year overall survival were 18–47%, 5.5–27.2%, and 39–57%, respectively [6–12].

Regardless of the nature of the primary tumor’s involvement with the urinary bladder, surgeons have to choose between performing partial versus total cystectomy to treat patients with this type of cancer. This choice is critical in terms of obtaining clear resection margins and achieving local disease control. However, the postoperative and survival outcomes of patients who undergo partial and total cystectomy have only been compared in a few studies of primary advanced colorectal cancer, each of which comprised a small number of patients.

Accordingly, this study aimed to investigate the short- and long-term outcomes of patients who underwent en bloc partial or total cystectomy for primary advanced colorectal cancer that was suspected of invading the urinary bladder.

## Methods

This study was approved by the Institutional Review Board of the National Cancer Center Hospital (Chiba, Japan), and included patients who underwent en bloc resection with partial or total cystectomy between 1993 and 2013 to treat locally advanced primary colorectal cancer that was suspected of invading the urinary bladder. All patients underwent preoperative evaluations for tumor-node-metastasis (TNM) staging, including colonoscopy, computed tomography, and magnetic resonance imaging. All treatment strategies were planned during multidisciplinary team conferences. For patients with rectal cancer who desired the retention of their anal function, it was considered possible to omit preoperative chemoradiotherapy if the circumferential resection margin was deemed clear. Preoperative cystoscopic examination was performed by a urologist if the patient had cloudy urine or hydronephrosis, or if invasion to the lumen of the urinary bladder was suspected based on the preoperative examination. Patients with recurrent tumors were excluded from this study, as were those in whom total cystectomy was performed solely because a lower rectal tumor had invaded the prostate.

All surgeries were performed via laparotomy by colorectal surgeons in conjunction with urologists; the goal of each surgery was en bloc resection. When performing partial cystectomy, the bladder defect was closed by 2 layers of running sutures. At our institution, the indication for total cystectomy is the presence of adhesion to/invasion into the vesical trigone, or an estimated urinary bladder capacity < 50 mL (not including patients who undergo ileal cystoplasty). While the extents of resection and reconstruction of the urinary bladder were decided preoperatively, the intended procedures were modified based on intraoperative findings in some cases.

All data were extracted retrospectively from medical records. The following variables were retrieved and analyzed: patient characteristics, presence of urinary tract infection (defined as significant bacteria in the urine culture), any preoperative therapy, preoperative staging, operative procedure, postoperative data (including urination status), pathological data, follow-up data, and long-term disease status. Tumor staging was performed according to the Union for International Cancer Control TNM classification. Local recurrence was defined as the detection of the disease in the pelvic cavity, whereas peritoneal dissemination was defined as the presence of intra-abdominal recurrence far from the pelvic cavity.

### Statistical analysis

The operative, postoperative, and pathological characteristics of the patients who underwent partial and total cystectomy (the P and T groups, respectively) were compared using the chi-square test (for categorical variables and proportions) or the Mann-Whitney *U* test (for continuous variables). The time to recurrence was measured from the date of surgery to that of first recurrence. Overall recurrence referred to both local recurrence and distant disease. Kaplan-Meier curves were used to estimate overall recurrence, local recurrence, and overall survival rates. Differences in survival were evaluated using the log-rank test. Cox proportional hazards modeling was used to estimate the independent associations between the characteristics of the patients and tumors on one hand and recurrence/survival on the other. Hazard ratios (HRs) are reported with 95% confidence intervals (95% CIs). All of the statistical analyses were performed using the Statistical Package for Social Sciences (SPSS version 22; IBM Corp., Armonk, NY). *P*-values less than 0.05 were considered statistically significant.

## Results

This study included 90 patients who underwent en bloc surgery and cystectomy for advanced primary colorectal cancer that was suspected of invading the urinary bladder; the P group comprised 72 patients who underwent en bloc partial cystectomy whereas the T group comprised the remaining 18 who underwent total cystectomy.

Of the 72 patients in the P group, 56 received full-thickness wall resection of the urinary bladder. Fifty-three of these 56 patients underwent primary closure of the defect of the urinary bladder, while the remaining 3 underwent en bloc partial cystectomy with ileal cystoplasty to compensate for low residual bladder capacity. In the P group, 17 patients underwent unilateral or bilateral ureterectomy with ureteric implantation. In the T group, the urinary tract was reconstructed as an ileal conduit in 14 patients and as an ileal neobladder in 4. The urinary bladder reconstruction procedures were not selected according to any definitive criteria but were performed according to the discretion of each individual surgeon.

Table 1 summarizes the patient characteristics and surgical outcomes. Preoperative metastatic tumors were significantly more common in the P group (*p* = 0.048). Because all concomitant metastatic lesions in this study were considered resectable, simultaneous or staged resections were performed for patients with concomitant distant metastases. The primary tumors were mainly located at either the sigmoid colon (*n* = 51) or the upper-to-middle rectum (*n* = 39); the distribution of primary tumor locations differed significantly between the 2 groups (*p* = 0.006). Five and 2 patients had received preoperative chemotherapy and chemoradiotherapy, respectively. Before en bloc resection or preoperative therapy, temporary stomas were initially created in 16 patients because of ileus or inflammation caused by the primary tumor. Seventy-five patients (83.3%) underwent anal preservation procedures. Patients in the T group had significantly longer operating times than those in the P group (median, 572 vs. 346 min) as well as significantly greater amounts of blood loss (median, 3092 vs. 1112 mL).

**Table 1:** Patient demographics and surgical outcomes

*Variables*	*Partial (n = 72)*	*Total (n = 18)*	*p value*
Age, y ^a^	63 (41–80)	61 (35–71)	0.146
Gender, Male/Female, n (%)	60 (83.3%) / 12 (16.7%)	15 (83.3%) / 3 (16.7%)	0.653
Location, n (%)			
Colon	46 (63.9%)	5 (27.8%)	0.006
Rectum	26 (36.1%)	13 (72.2%)	
Preoperative M stage, n (%)			
M0	53 (73.6%)	17 (94.4%)	0.048
M1	19 (26.4%)	1 (5.6%)	
Preoperative therapy, n (%)			
Chemotherapy	5 (6.9%)	0 (0%)	
Chemoradiotherapy	1 (1.4%)	1 (5.5%)	
Preoperative WBC count ^a^	8000 (3500–18,000)	8100 (5900–15,200)	0.515
Preoperative UTI, n (%)	12 (16.7%)	5 (27.8%)	0.224
Preoperative stoma creation, n (%)	12 (16.7%)	4 (22.2%)	0.4
Operative procedure, n (%)			
Sigmoidectomy	31 (43.1%)	1 (5.5%)	
High anterior resection	17 (23.6%)	1 (5.5%)	
Low anterior resection	19 (26.4%)	3 (16.8%)	
Intersphincteric resection	0 (0%)	1 (5.5%)	
Hartmann	5 (6.9%)	0 (0%)	
Total pelvic exenteration	0 (0%)	12 (66.7%)	
Operative time, min ^a^	346 (120–765)	572 (369–813)	< 0.001
Blood loss, mL ^a^	1112 (88–6194)	3092 (779–9000)	< 0.001

*WBC* white blood cell, *UTI* urinary tract infection

^a^ Median (range) shown

Table 2 shows the postoperative and pathological outcomes. The postoperative overall complication rate was significantly higher in the T group (94.4%) than in the P group (51.4%). One patient in the P group died during hospitalization because of severe, extensive infection at the deep surgical site. Postoperative hospital stays were significantly shorter for patients in the P group (median, 21 days) than they were for those in the T group (median, 29 days). Despite a high frequency of postoperative complications, only 4 patients were unable to achieve a ureteral catheter-free state because of urinary tract leakage and fistula formation. There were no statistically significant differences between the P and T groups in terms of pathological bladder involvement, pathological nodal status, or tumor size. Postoperative chemotherapy was administered to 22 patients (24.4%).

**Table 2:** Postoperative and pathological outcomes

*Variables*	*Partial (n = 72)*	*Total (n = 18)*	*p value*
Complications, n (%)	37 (51.4%)	17 (94.4%)	< 0.001
Urinary tract leakage	17 (23.6%)	5 (27.7%)	
Intestinal AL	4 (5.6%)	2 (11.1%)	
Ileus	1 (1.4%)	0 (0%)	
Urinary tract infection	4 (5.6%)	8 (44.4%)	
Surgical site infection	6 (8.3%)	6 (33.3%)	
Intrapelvic abscess	1 (1.4%)	4 (22.2%)	
Gas gangrene	1 (1.4%)	0 (0%)	
Complications of CD ≧ 3a, n (%)	15 (20.8%)	3 (16.7%)	0.49
Mortality, n (%)	1 (1.4%)	0 (0%)	
Postoperative hospital stay, day	21 (11–241)	29 (20–241)	0.001
Spontaneous urination, n (%)	68 (94.4%)	–	
Periods of catheter insertion, day	14 (2–624)	–	
Bladder involvement, n (%)	26 (36.1%)	7 (38.9%)	0.827
N stage, n (%)			
N0	41 (56.9%)	10 (55.6%)	0.915
N1	24 (33.3%)	6 (33.3%)	
N2	7 (9.8%)	2 (11.1%)	
Tumor size, mm ^a^	90 (30–182)	80 (31–140)	0.192
Tumor Grade, n (%)			
WD	6 (8.3%)	4 (22.2%)	0.075
MD	61 (84.7%)	10 (55.6%)	
PD or mucinous	5 (7.0%)	4 (22.2%)	
Postoperative chemotherapy, n (%)	19 (26.4%)	3 (16.7%)	0.25

*AL* anastomotic leakage, *CD* Clavien-Dindo, *WD* Well differentiated. *MD* Moderately differentiated, *PD* Poorly differentiated

^a^ Median (range) shown

**Fig. 1:**
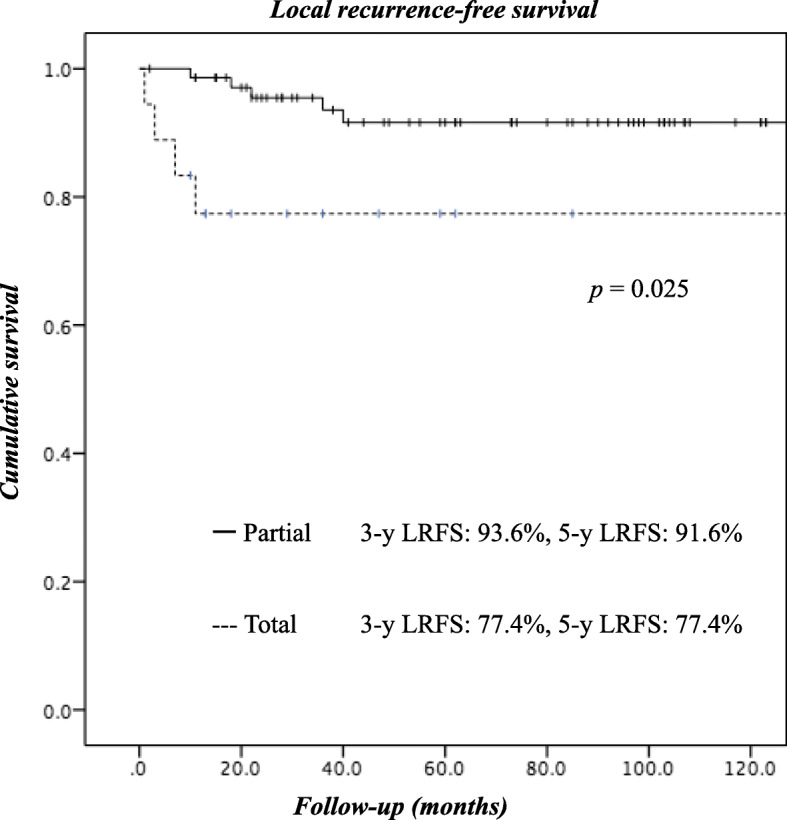
Kaplan-Meier analysis of local recurrence-free survival (LRFS). Shown are LRFS curves for patients who underwent partial cystectomy vs. those who underwent total cystectomy (log-rank test, *p* = 0.025)

**Fig. 2:**
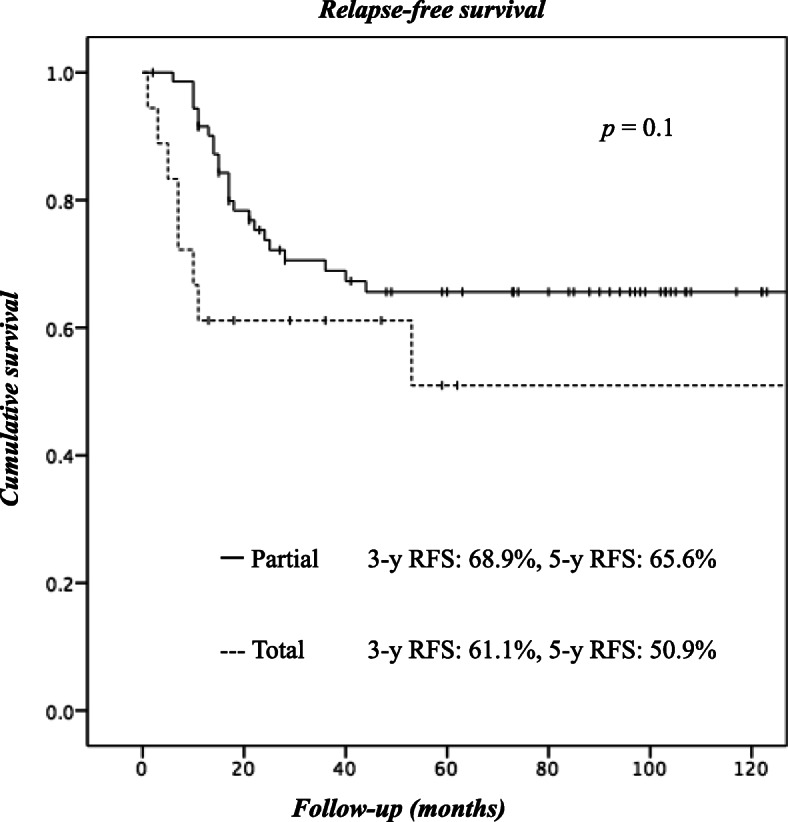
Kaplan-Meier analysis of relapse-free survival (RFS). Shown are RFS curves for patients who underwent partial cystectomy vs. those who underwent total cystectomy (log-rank test, *p* = 0.1)

**Fig. 3:**
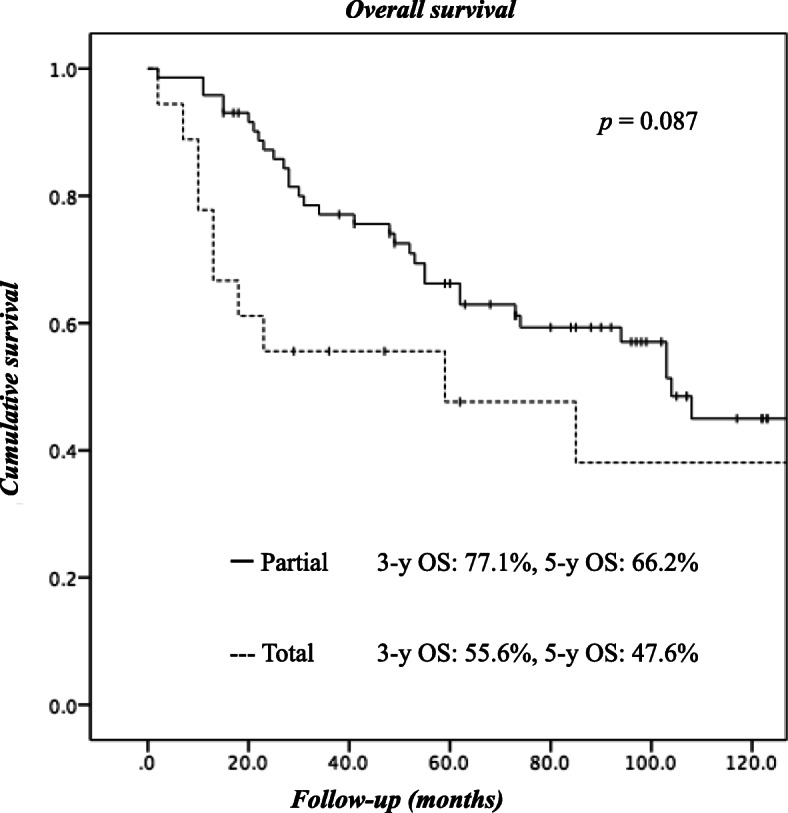
Kaplan-Meier analysis of overall survival (OS). Shown are OS curves for patients who underwent partial cystectomy vs. those who underwent total cystectomy (log-rank test, *p* = 0.087)

With a median follow-up of 62 months after surgery, distant metastasis and local recurrence were observed in 24 and 9 patients, respectively (Table 3). Recurrence of peritoneal dissemination was observed in only 4.6% of the patients in the P group. Furthermore, the local recurrence rate was lower in the P group (6.9%) than in the T group (22.2%). The 5-year local recurrence-free, relapse-free, and overall survival rates of all the patients were 88.6, 62.9, and 62.5%, respectively. The local recurrence-free survival curves of the 2 groups were significantly different, with worse outcomes observed in the T group (Fig. 1). Although the Kaplan-Meier survival curves for relapse-free survival (Fig. 2) and overall survival (Fig. 3) did not differ significantly between the groups, the outcomes of patients in the T group tended to be worse than those for patients in the P group.

**Table 3:** Recurrence patterns

		Type of Cystectomy	
	Total (*n* = 90)	Partial (*n* = 72)	Total (*n* = 18)
Overall recurrence, n (%)	31 (34.4%)	23 (31.9%)	8 (44.4%)
Local recurrence	9 (10%)	5 (6.9%)	4 (22.2%)
Peritoneal dissemination	3 (3.3%)	3 (4.2%)	0 (0%)
Liver	12 (13.3%)	9 (12.5%)	3 (16.7%)
Lung	10 (11.1%)	7 (9.7%)	3 (16.7%)
Distant lymph node	4 (4.4%)	3 (%)	1 (5.6%)
Brain	2 (2.2%)	2 (2.8%)	0 (0%)

On multivariate Cox regression analyses using the P group as the reference, patients in the T group were significantly and independently associated with worse local recurrence-free survival (HR, 4.0; 95% CI, 1.1–15.0), relapse-free survival (HR, 2.9; 95% CI, 1.2–6.9), and overall survival (HR, 2.1; 95% CI, 1.0–4.3) (Table 4). The presence of synchronous metastatic diseases was also independently and significantly associated with poorer relapse-free survival (HR, 3.2; 95% CI, 1.4–7.3) and poorer overall survival (HR, 3.1; 95% CI, 1.6–6.3). Furthermore, pathological lymph node metastasis was significantly and independently associated with worse relapse-free survival (HR, 2.6; 95% CI, 1.3–5.5).

**Table 4:** Cox proportional hazards analyses

	Local recurrence-free survival			Relapse-free survival			Overall survival		
	Uni	Multi		Uni	Multi		Uni	Multi	
*Variable*	*P value*	*Hazard ratio (95% CI)*	*P value*	*P value*	*Hazard ratio (95% CI)*	*P value*	*P value*	*Hazard ratio (95% CI)*	*P value*
Gender	0.536			0.620			0.187		
Age > 65 years	0.606			0.846			0.650		
Colon vs rectum	0.332			0.276			0.379		
Synchronous metastasis	0.359			0.028	3.215 (1.413–7.314)	0.005	0.102	3.143 (1.563–6.323)	0.001
Total cystectomy	0.075	4.022 (1.076–15.034)	0.039	0.318	2.875 (1.204–6.866)	0.017	0.246	2.097 (1.033–4.260)	0.041
BL > 1500 mL	0.162			0.957			0.686		
Postoperative complication	0.061			0.526			0.796		
Pathological bladder involvement	0.189			0.225			0.705		
Pathological LN metastasis	0.332			0.013	2.633 (1.253–5.531)	0.011	0.691		

*Uni* univariate analysis, *Multi* multivariate analysis, *BL* blood loss, *LN* lymph node

## Discussion

Our study demonstrated that en bloc total cystectomy for advanced colorectal cancer is associated with a longer operating time, greater blood loss, a higher postoperative complication rate, and a longer hospital stay than en bloc partial cystectomy. Postoperative urinary function was achieved in 94.4% of the patients in the P group. Furthermore, oncologic outcomes were generally worse in T-group patients than in their P-group counterparts.

Multivisceral resection for advanced colorectal cancer has been investigated in several previous studies. A systematic review by Mohan et al. revealed that the urinary bladder was the most commonly involved organ overall (53.2% of patients underwent urinary bladder resections) and that 54.1% of the resected specimens were found to exhibit tumor invasion [13]. Moreover, the reported rates of overall complications, mortality, and 5-year overall survival were 41.5, 4.2, and 50.3%, respectively. Our patients’ postoperative outcomes and 5-year overall survival rates were generally consistent with those reported by Mohan et al.

Among the multivisceral resection methods used to treat advanced colorectal cancer, total pelvic exenteration (TPE) is the most invasive and least function-preserving. In a previous systematic review of 23 studies that comprised 1049 patients who underwent TPE, the overall complication and mortality rates were 57 and 2.2%, respectively [14]. Ten (55.6%) of the 18 patients who underwent total cystectomy in the current study underwent TPE, all of whom developed several complications including 1 who required invasive intervention. However, none of the patients who underwent TPE in our study died because of such postoperative complications. Regardless of whether TPE was performed, total cystectomy was clearly associated with a higher overall complication rate than partial cystectomy. This may be attributed not only to wider resections, but also to the longer operative times and greater estimated blood loss volumes among patients undergoing total cystectomy. Although only 13.9% of all patients underwent TPE, this invasive surgery was invariably associated with the high complication rate that was observed.

The overall postoperative complication rate in our study was high (60%), especially among patients who underwent TPE (100%). Our findings suggest that laparotomy, which was used in all our surgeries, may be associated with a high complication rate when applied for locally advanced colorectal cancer that adheres to or invades other organs. In recent years, laparoscopic procedures have become acceptable treatment options for these tumors. Previous studies revealed that laparoscopic surgeries are associated with shorter operative times, lower blood loss volumes, and shorter postoperative hospital stays than are conventional open surgeries [15, 16]. However, a phase 3, randomized controlled trial that included 1057 patients found that laparoscopic surgery tended to be associated with worse survival than open surgery when treating cT4 disease [17]. As such, it remains controversial whether laparoscopic or open surgery should be favored for locally advanced colorectal cancer that has adhered to or invaded other organs.

Feinberg et al. [18] performed a systematic review of oncologic outcomes following laparoscopic versus open resection of pathological T4 colon tumors and found no significant differences in the rates of overall survival, disease-free survival, or positive surgical margins between the 2 groups. Furthermore, a recent study found that robot-assisted multivisceral resection is advantageous for patients with rectal cancer, although the study included only a small number of patients [19]. Only a limited number of studies have investigated laparoscopic bladder resection for advanced colorectal cancer. Nagasue et al. [15] reported that patients who underwent laparoscopic bladder resection accounted for only 10% of all those who underwent any type of laparoscopic multivisceral resection. On the other hand, patients who underwent conventional open bladder resection accounted for 31.8% of all those who underwent open multivisceral resection [15]. Because laparoscopic en bloc cystectomy for advanced colorectal cancer appears to be technically difficult, the indications for this procedure should be determined carefully.

Local recurrence was observed in 10% of all the patients in our study, which is generally similar to or lower than the rates described in other published reports [6, 8–12] (Table 5). However, local recurrence and overall survival rates after total cystectomy were significantly worse than those after partial cystectomy. Gao et al. [10] reported that survival after surgery for colorectal cancer is not influenced by the need to excise part or all of the urinary bladder. Balbay et al. reported that the rates of local recurrence (14% vs. 17%) and 3-year survival (49% vs. 39%) did not differ significantly between their partial and total cystectomy groups, although a tendency toward slightly worse outcomes was observed in the latter [6]. Previous studies did not clearly conclude that en bloc total cystectomy impairs oncologic outcomes. Nevertheless, our results may indicate that advanced colorectal cancer requiring en bloc total cystectomy should be treated with a multimodal approach to improve oncologic outcomes, such as by combining surgical treatment with neoadjuvant chemotherapy or chemoradiation.

**Table 5:** Reports on en bloc cystectomy for colorectal cancer

Study	n (partial / total)	Preoperative CRT (%)	Complication (%)	Local rec (%)	Distant rec (%)	Survival (%)
Balbay et al. [6]	81 (35 / 46) ※rec cases: 35	60.5%	33.3%	17%	NA	49% (3-y OS)
Carne et al. [8]	53 (45 / 4) ※no resection: 4	NA	NA	22.6%	NA	NA
Winter et al. [9]	63 (53 / 10)	36.5%	18%	14%	NA	57% (5-y OS)
Gao et al. [10]	33 (28 / 5)	NA	48.5%	27.3%	NA	39% (5-y OS)
Li et al. [11]	72 (58 / 14)	0%	47%	9.7%	19.4%	50% (5-y OS)
Luo et al. [12]	84 (84 / 0)	0%	NA	8.3% ^a^	25%	NA
Our cases	90 (72 / 18)	2.2%	60%	10%	28.9%	63% (5-y OS)

*rec* recurrence, *CRT* chemoradiotherapy, *OS* overall survival

^a^ Included only the cases of urinary bladder recurrence

In our study, preoperative chemotherapy and chemoradiotherapy were administered to only 5.6 and 2.2% of all patients, respectively. One patient in each of the P and T groups received neoadjuvant chemoradiation therapy; the former developed urinary tract leakage while the latter had a refractory deep surgical site infection. However, neither patient developed local recurrence during the study, and both achieved extended survival times (98 and 47 months, respectively). Oledzki et al. [7] reported that the local recurrence rate in patients who received preoperative chemoradiotherapy followed by total cystectomy for rectal cancer was only 5%. Furthermore, a recent study by Cukier et al. on preoperative chemoradiotherapy followed by multivisceral resection for colon cancer revealed a local recurrence rate of only 6% and a 3-year overall survival rate of 85.9% [20]. Because these studies demonstrated the achievement of good local control, preoperative chemoradiotherapy should become a more widely adopted treatment option for controlling locally advanced colorectal cancers that require total cystectomy. However, administering preoperative chemoradiotherapy to these patients may carry the risk of worse postoperative short-term outcomes, especially when urinary tract reconstruction is required.

Of note, there were several limitations to our study. First, the study comprised an analysis of retrospective data from a single center. Second, imaging technologies, surgical techniques (such as laparoscopy), and postoperative management methods have all undergone marked improvements in recent years, thereby influencing postoperative and oncologic outcomes and possibly rendering our findings not representative of present-day clinical practice. Despite these limitations, our results ought to be considered valuable and applicable owing to the relatively large number of patients as well as our use of a standardized organ-preserving strategy.

## Conclusions

The results of this study show that locally advanced colorectal cancer requiring en bloc total cystectomy was associated with worse clinical and oncological outcomes. Compared to patients in the T group, those in the P group whose tumors did not infiltrate the bladder neck or trigone had high urinary function preservation rates, lower frequencies of local recurrence, and improved survival. Because en bloc total cystectomy was associated with impairments in both short- and long-term outcomes, multimodal treatments and less invasive surgical techniques should be considered for tumors that require en bloc total cystectomy.

## Data Availability

The datasets used and/or analyzed during the current study available from the corresponding author on reasonable request.

## Abbreviations

CI: Confidence interval
HR: Hazard ratio
LRFS: Local recurrence-free survival
OS: Overall survival
RFS: Relapse-free survival
TNM: Tumor-node-metastasis
TPE: Total pelvic exenteration
